# Angiographic severity in acute coronary syndrome patients with and without standard modifiable risk factors

**DOI:** 10.3389/fcvm.2022.934946

**Published:** 2022-07-22

**Authors:** Andreas S. Papazoglou, Ioannis T. Farmakis, Stefanos Zafeiropoulos, Dimitrios V. Moysidis, Efstratios Karagiannidis, Nikolaos Stalikas, Anastasios Kartas, Konstantinos Stamos, Georgios Sofidis, Ioannis Doundoulakis, Georgios Giannopoulos, George Giannakoulas, Georgios Sianos

**Affiliations:** ^1^Department of Cardiology, AHEPA University Hospital, Aristotle University of Thessaloniki, Thessaloniki, Greece; ^2^Athens Naval Hospital, Athens, Greece; ^3^First Department of Cardiology, School of Health Sciences, National and Kapodistrian University of Athens, Athens, Greece; ^4^Third Department of Cardiology, Medical School, Hippocration General Hospital, Aristotle University of Thessaloniki, Thessaloniki, Greece

**Keywords:** acute coronary syndrome, angiographic severity, SYNTAX score, standard modifiable cardiovascular disease risk factors, myocardial infarction

## Abstract

**Background:**

Routine coronary artery disease (CAD) secondary prevention strategies target standard modifiable cardiovascular risk factors (SMuRFs), which include: diabetes mellitus, dyslipidemia, hypertension, and smoking. However, a significant proportion of patients with acute coronary syndrome (ACS) present without any SMuRFs. The angiographic severity of disease in this population has not yet been investigated.

**Methods:**

After propensity score matching of patients without SMuRFs and patients with ≥1 SMuRFs (ratio 1:3), we used zero-inflated negative binomial regression modeling to investigate the relationship of SMuRF-less status with the angiographic severity of CAD, as measured by the SYNTAX score. Survival analysis was performed to investigate differences in all-cause mortality at 30 days and at the end of follow-up period.

**Results:**

We analyzed 534 patients presenting with ACS who underwent coronary angiography. Of them, 56 (10.5%) presented without any SMuRF. After propensity score matching, the median SYNTAX score was 13.8 (IQR 0–22.1) in 56 SMuRF-less patients and 14 (IQR 5–25) in 166 patients with ≥1 SMuRFs. SMuRF-less status was associated with increased odds of zero SYNTAX score [zero-part model: odds ratio = 2.11, 95% confidence interval (CI): 1.03–4.33], but not with decreased SYNTAX score among patients with non-zero SYNTAX score (count-part model: incidence rate ratio = 0.99, 95% CI: 0.79–1.24); the overall distribution of the SYNTAX score was similar between the two groups (*p* = 0.26). The 30-day risk for all-cause mortality was higher for SMuRF-less patients compared to patients with ≥1 SMuRFs [hazard ratio (HR) = 3.58, 95% CI: 1.30–9.88]; however, the all-cause mortality risk was not different between the two groups over a median 1.7-year follow-up (HR = 1.72, 95% CI: 0.83–3.57).

**Conclusion:**

Among patients with ACS, the absence of SMuRFs is associated with increased odds for non-obstructive CAD and with increased short-term mortality rates.

## Introduction

Annually, millions of people suffer an acute coronary syndrome (ACS) driven by an atherosclerosis-related coronary artery narrowing or occlusion (1). The majority present with at least one standard modifiable cardiovascular risk factor (SMuRF) (2), namely diabetes mellitus (DM), dyslipidemia, hypertension, and smoking, which are considered to be key drivers of coronary artery disease (CAD). These SMuRFs constitute the central elements of the Framingham risk score and other validated algorithms, thereby, driving evidence-based guideline recommendations, including the latest European Society of Cardiology guidelines for cardiovascular disease prevention in clinical practice (3, 4). Traditionally, primary and secondary CAD prevention has been oriented toward timely identification and targeted treatment of the SMuRFs.

However, a growing body of research now recognizes a significant proportion of patients presenting with ACS without having any SMuRF (SMuRF-less status) (5–8). The proportion of SMuRF-less patients seems to be rising during the last two decades and has been reported to be up to 20% (5). Recent studies have also investigated the prognostic impact of the SMuRF-less status on the clinical course of ACS, yielding contradictory data on patients’ short- and long-term survival (5–7, 9). Nevertheless, differences in intrinsic characteristics of the ACS and especially in coronary angiographic characteristics in SMuRF-less patients, as compared to the general ACS patient population, have not been studied yet.

To that end, our study aims to investigate associations of the SMuRF-less status with CAD angiographic severity, as assessed with the SYNTAX score, and evaluate the association of the SMuRF-less status with short- and long-term mortality rates in a cohort of well-characterized ACS patients.

## Materials and methods

### Study design

This is a *post hoc* analysis conducted among patients with ACS, enrolled in the *Correlation of the severity of Coronary Artery Disease with patients’ metabolic profile* (CorLipid) trial (ClinicalTrials.gov, identification number: NCT04580173). In brief, the CorLipid trial aimed: (1) to investigate the metabolic fingerprint of patients undergoing coronary angiography in AHEPA University Hospital of Thessaloniki, Greece, and (2) to develop metabolomics-based predictive models for the prediction of CAD severity. The protocol and main outcomes of the trial have been already published (10, 11). The study protocol was approved by the institutional review board (AHEPA University Hospital IRB registration number 12/13-06-2019), and the trial was conducted in compliance with the principles of the Declaration of Helsinki.

### Patient population

Eligible for the current analysis were deemed all CorLipid participants who presented with ACS, underwent coronary angiography, and provided written informed consent for study participation prior to coronary angiography. Exclusion criteria included prior medical history of CAD, cardiopulmonary arrest at presentation, severe concomitant disease with life expectancy less than 1 year, or no recorded information regarding history of hypertension, DM, dyslipidemia, and smoking status.

### Data sources and definition

Data were extracted from the CorLipid database for the time range of July 2019 (first patient enrollment) to May 2021 (last patient enrollment). The database included variables describing medical history, baseline clinical, laboratory, and angiographic characteristics.

The SMuRFs (DM, dyslipidemia, hypertension, and smoking) were all defined as binary variables, recorded at patient enrollment. Patients with DM, dyslipidemia and hypertension were defined as those meeting at least one of the following criteria: (a) administration of anti-diabetic, anti-hyperlipidemic or anti-hypertensive medication, respectively; (b) diagnostic code of DM, dyslipidemia or hypertension (International Classification of Diseases-10) in at least one hospital admission; or (c) history of medical illnesses as provided by the patients. Smoking history was defined as current self-reported use of tobacco products.

Coronary angiographic images of every patient were independently evaluated by two experienced interventional cardiologists (GSi and GSo), blinded to patient clinical characteristics and outcomes, who calculated the SYNTAX score. SYNTAX score constitutes an angiographic grading tool to determine CAD complexity. It is derived from summarizing the points assigned to each individual lesion identified in the coronary arterial tree with at least 50% diameter narrowing in vessels of more than 1.5 mm diameter (12).

Follow-up data were acquired until November 2021 through telephonic or in-person interviews with study participants. The vital status of all patients was additionally verified through the Greek Civil Registration System.

### Outcomes of interest

The primary study outcome was the association of the SMuRF-less status with the angiographic severity of CAD, as assessed with the SYNTAX score. A secondary study outcome was to compare the risk of all-cause mortality in patients with and without SMuRFs according to both short- and long-term patients’ follow-up (at 30 days and using the longest available median 1.7-year follow-up, respectively).

### Statistical analysis

#### Descriptive analyses

Categorical variables are summarized as frequencies with percentages, and continuous variables as medians with interquartile ranges (IQR = Q3 − Q1). The Pearson Chi-square or Fisher’s exact test were used for comparison between categorical variables, whereas continuous variables were compared using the Wilcoxon rank-sum test.

#### Handling of missing data

Missing observations were regarded as missing at random and were dealt with the use of multiple imputation; a predictive mean matching method with 50 iterations was used to create 5 complete imputation datasets.

#### Propensity score matching

We used propensity score matching without replacement to adjust for confounding. Patients without any SMuRFs were matched with patients with ≥1 SMuRFs on a 1:3 ratio. We used greedy nearest neighbor propensity score matching without replacement with a propensity score generated by logistic regression with the use of age, sex, family history of CAD, body mass index (BMI), estimated glomerular filtration rate (eGFR), and ST-elevation myocardial infarction (STEMI) occurrence at the index event as covariates. Covariate balance was assessed with the use of a Love plot, and standardized mean differences below 0.1 for all covariates indicated adequate balance.

#### Regression modeling for the association of standard modifiable cardiovascular risk factor-less status with the SYNTAX score

Aiming to avoid assessing the SYNTAX score as an arbitrarily defined categorical variable, we assessed its distribution as a continuous variable in our analysis. Specifically, because of the excessive presence of zero values in the SYNTAX score distribution, a zero-inflated binomial regression model was utilized to associate the SMuRF-less status with the SYNTAX score in the matched population. This model separated the regression into two distinct parts: (a) a first part to predict whether a patient is more likely to present with a zero or non-zero SYNTAX score (*zero-part* model); and (b) a second part to predict the SYNTAX score in patients with a non-zero SYNTAX score (*count-part* model). Odds ratios (ORs) and incidence rate ratios (IRRs) with corresponding 95% confidence intervals (CIs) are reported to summarize the effect of the SMuRF-less status on the SYNTAX score in the two parts, respectively.

#### Survival analysis

The Kaplan–Meier estimator was used to plot survival curves for the all-cause mortality outcome from the date of admission up to 30 days and up to the total available follow-up stratified by the absence or not of SMuRFs (matched population). A Cox regression analysis was performed in the matched population to report hazard ratios (HRs) with corresponding 95% CIs for the risk of early- and long-term all-cause mortality in patients without SMuRFs.

All analyses were conducted in R (the R Project for Statistical Computing, version 3.6.3) with the use of *tidyverse*, *mice*, *matchIt*, *pscl*, and *survival* packages. A two-sided *p*-value of less than 0.05 was considered statistically significant.

## Results

### Patient characteristics

Of the 958 patients enrolled in the CorLipid study, a total of 534 (55.7%) presented with ACS and their data were utilized for the current analysis with a median follow-up of 1.7 years (study flowchart presented in Supplementary Figure 1). Median age was 63 (IQR = 17) years, 129 (24.2%) were females and 41.7% presented with an STEMI. Of 534 patients with ACS, 56 (10.5%) presented without any SMuRF. SMuRF-less patients presented more commonly with dyspnea rather than with chest pain, were less obese and had lower low density lipoprotein cholesterol (LDL-C) and eGFR values than patients with ≥1 SMuRFs (Tables 1, 2).

**TABLE 1:** Baseline clinical and demographic characteristics of the included population.

Characteristic	Overall patients with ACS (*N* = 534)	Patients with at least one SMuRF (*N* = 478)	Patients without SMuRFs (*N* = 56)	*P*-vaue^1^
Age, years, median (IQR)	63 (17)	63 (17)	66 (24)	0.6
Sex, male, *n* (%)	405 (75.8%)	366 (76.6%)	39 (69.6%)	0.3
BMI, kg/m^2^, median (IQR)*	27.8 (5.3)	27.9 (5.2)	26.2 (5.9)	**0.010**
Hypertension, *n* (%)	285 (53.4%)	285 (59.6%)	0 (0.0%)	**<0.001**
Diabetes mellitus, *n* (%)	131 (24.5%)	131 (27.4%)	0 (0.0%)	**<0.001**
Dyslipidemia, *n* (%)*	170 (31.9%)	170 (35.6%)	0 (0.0%)	**<0.001**
Smoking, *n* (%)	285 (53.4%)	285 (59.6%)	0 (0.0%)	**<0.001**
Family history of CAD, *n* (%)*	109 (20.5%)	101 (21.2%)	8 (14.3%)	0.2
Previous stroke, *n* (%)*	14 (2.6%)	14 (2.9%)	0 (0.0%)	0.4
PAD, *n* (%)	25 (4.7%)	24 (5.0%)	1 (1.8%)	0.5
Atrial fibrillation, *n* (%)	43 (8.1%)	36 (7.5%)	7 (12.5%)	0.2
CKD, *n* (%)	30 (5.6%)	27 (5.6%)	3 (5.4%)	1
COPD, *n* (%)	28 (5.2%)	27 (5.6%)	1 (1.8%)	0.3
Severe aortic stenosis, *n* (%)	6 (1.1%)	4 (0.8%)	2 (3.6%)	0.12
Heart failure, *n* (%)	12 (2.2%)	12 (2.5%)	0 (0.0%)	0.6
ACS type, *n* (%)				0.8
NSTEMI	169 (31.8%)	150 (31.5%)	19 (33.9%)	
STEMI	222 (41.7%)	201 (42.2%)	21 (37.5%)	
Unstable angina	141 (26.5%)	125 (26.3%)	16 (28.6%)	
Chest pain, *n* (%)	432 (81.1%)	396 (83.0%)	36 (64.3%)	<0.001
Dyspnea, *n* (%)	91 (17.1%)	74 (15.5%)	17 (30.4%)	**0.005**
Easy fatigue, *n* (%)	29 (5.4%)	28 (5.9%)	1 (1.8%)	0.3

^1^Wilcoxon rank sum test; Pearson’s Chi-squared test; Fisher’s exact test.

*Missing values for the variable in less than 5% of the study sample. BMI, body mass index; CAD, coronary artery disease; PAD, peripheral artery disease; CKD, chronic kidney disease; COPD, chronic obstructive pulmonary disease; ACS, acute coronary syndrome; (N)STEMI, (non-)ST-elevated myocardial infarction; GFR, glomerular filtration rate. The bold values represent statistically significant outcomes (p-value < 0.05).

**TABLE 2:** Baseline clinical, laboratory, electrocardiographic, and angiographic characteristics of the included population.

Characteristic	Overall patients with ACS (*N* = 534)	Patients with at least one SMuRF (*N* = 478)	Patients without SMuRFs (*N* = 56)	*P*-vaue^1^
Heart rate, median (IQR)*	76 (15)	76 (15)	75 (19)	0.9
Systolic blood pressure, median (IQR)	129 (29)	129 (29)	128 (28)	0.5
Diastolic blood pressure, median (IQR)	79 (14)	78 (14)	80 (18)	0.5
SYNTAX score, median (IQR)	15 (18)	15 (17)	14 (22)	0.3
GRACE score, median (IQR)*	117 (52)	115 (50)	126 (75)	0.3
QRS duration ms, median (IQR)*	95 (10)	95 (10)	92 (20)	0.062
ST-T changes in ECG, *n* (%)	340 (63.7%)	306 (64.0%)	34 (60.7%)	0.6
Q wave in ECG, *n* (%)*	98 (20.2%)	87 (20.0%)	11 (22.4%)	0.7
High-sensitivity troponin-T, median (IQR)*	247 (1,266)	247 (1,222)	248 (1,751)	0.7
GFR, median (IQR)*	96 (39)	98 (39)	86 (44)	**0.008**
Total cholesterol, median (IQR)*	158 (61)	159 (63)	147 (54)	0.053
Triglycerides, median (IQR)*	129 (80)	129 (78)	122 (94)	0.7
LDL-cholesterol, median (IQR)*	89 (54)	91 (55)	78 (34)	**0.016**
HDL-cholesterol, median (IQR)*	39 (14)	39 (13)	39 (18)	0.8

^1^Wilcoxon rank sum test; Pearson’s Chi-squared test; Fisher’s exact test.

*Missing values for the variable in less than 5% of the study sample. BMI, body mass index; CAD, coronary artery disease; PAD, peripheral artery disease; CKD, chronic kidney disease; COPD, chronic obstructive pulmonary disease; ACS, acute coronary syndrome; (N)STEMI, (non-)ST-elevated myocardial infarction; GFR, glomerular filtration rate. The bold values represent statistically significant outcomes (p-value < 0.05).

### Propensity-score–matched study samples

Among the 534 patients presenting with ACS, a total of 224 were matched according to the propensity score matching procedure (in a 1:3 ratio of patients without SMuRFs to patients with ≥1 SMuRFs); the baseline covariates had a minimal proportion of missing values (Supplementary Figure 2) and were well balanced in the propensity-score–matched samples (Supplementary Table 1 and Supplementary Figure 3).

### Angiographic severity of coronary artery disease according to the presence of standard modifiable cardiovascular risk factors

**FIGURE 1:**
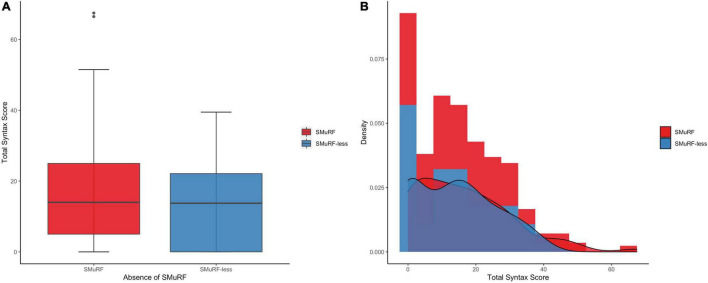
SYNTAX score distribution by the presence of SMuRFs. **(A)** The boxplots depict almost equal median SYNTAX score among patients with and without SMuRFs. **(B)** The histogram indicates the higher proportion of zero SYNTAX score in SMuRF-less patients. SMuRF, standard modifiable risk factors.

Among the SMuRF-less patients, the median SYNTAX score was 13.8 (IQR = 0–22.1), whereas in patients with ≥1 SMuRFs the median SYNTAX score was 14 (IQR = 5–25; Figure 1). In total, 19.2% of matched patients had a SYNTAX score of zero (28.6% among SMuRF-less patients and 16.1% among patients with ≥1 SMuRFs). The zero-part of the zero-inflated negative binomial regression model demonstrated that the SMuRF-less status was significantly associated with an increased probability for zero SYNTAX score (OR = 2.11, 95% CI 1.03–4.33). In the count-part of the model there was no significant association between the SMuRF-less status and the SYNTAX score (IRR = 0.99, 95% CI 0.79–1.24) among patients with non-zero SYNTAX score. There was no difference in the overall distribution of the SYNTAX score between the two groups (*p* = 0.26).

### Post-acute coronary syndrome mortality rates according to the presence of standard modifiable cardiovascular risk factors

**FIGURE 2:**
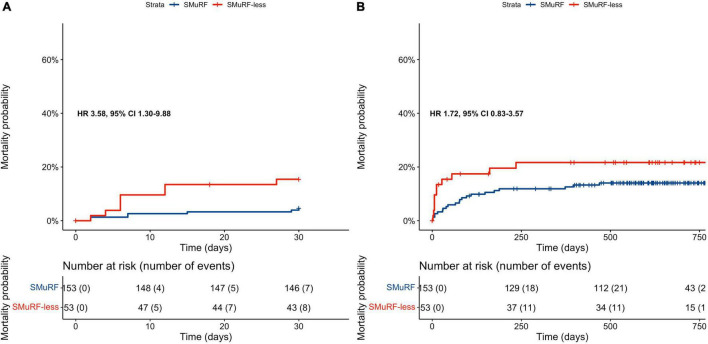
Kaplan–Meier curves depending on the presence of SMuRFs for: **(A)** 30-day all-cause mortality rates, **(B)** long-term all-cause mortality rates. SMuRF-less patients had significantly higher incidence of 30-day all-cause mortality; however, long-term mortality rates did not differ significantly among patients with and without SMuRFs. Both survival analyses included 206/224 matched participants since follow-up data were not available for the remaining ones.

The survival analyses included 206 matched participants since follow-up data were not available for 18 subjects. The 30-day all-cause mortality rate of patients with complete follow-up was significantly higher in SMuRF-less patients than in patients with ≥1 SMuRFs (cumulative incidence rates: 15.4 vs. 4.6%; HR = 3.58, 95% CI: 1.30–9.88, Figure 2A and Table 3).

**TABLE 3:** Clinical follow-up outcomes according to zero or ≥1 standard modifiable risk factors.

Outcome	Matched patients with at least one SMuRF (*N* = 168)	Matched patients without SMuRFs (*N* = 56)
Death from any cause at 30 days, *n* (cumulative incidence rate %)	7 (4.6%)	8 (15.4%)
Hazard ratios (95% confidence intervals)	Reference	HR = 3.58 (95% CI: 1.30–9.88)
Death from any cause at a median 1.7-year follow up, *n* (cumulative incidence rate %)	21 (14%)	11 (21.7%)
Hazard ratios (95% confidence intervals)	Reference	HR = 1.72 (95% CI: 0.83–3.57)

Over a median 1.7-year follow-up of study participants, all-cause mortality rates did not differ significantly among SMuRF-less patients and patients with ≥1 SMuRFs (cumulative incidence rates: 21.7 vs. 14%; HR = 1.72, 95% CI: 0.83–3.57, Figure 2B and Table 3).

## Discussion

In this analysis, roughly 1 out of 10 ACS patients presented without any SMuRF. These patients had an almost twofold increased probability of zero SYNTAX score as compared with patients with ≥1 SMuRFs. However, there was no difference in the overall SYNTAX distribution. The short-term risk of all-cause mortality was significantly higher in SMuRF-less patients than in patients with ≥1 SMuRFs. Nevertheless, long-term mortality rates did not differ between the two groups.

The hypothesis that CAD might be primarily driven by other non-conventional risk factors beyond the four well-established ones has been already stated since the late 2000s (13, 14). A patient-level meta-analysis of 122,458 patients with CAD, published in 2003, reported that 17% of them did not have any conventional risk factors (14). The terms “SMuRF” and “SMuRF-less” have been coined in 2017 by Vernon et al. (6). Patients presenting with ACS without any SMuRFs have been recognized as a significant and separate clinical entity since their proportion has shown increasing trends during the last decades (5, 15, 16). The prevalence of SMuRF-less status in cardiovascular disease seems to range from 3 to 27% depending on the studied population (2, 5–7, 9, 14, 17–19). Our findings concur with existing literature, demonstrating that roughly 1 out of 10 patients who presented with ACS without any prior history of cardiovascular disease had no SMuRFs (2, 7, 17, 20, 21).

To the best of our knowledge, this is the first study examining the angiographic severity of CAD in ACS patients with and without SMuRFs. Despite the increased odds of non-obstructive CAD (zero SYNTAX score) in ACS patients presenting without SMuRFs, the severity of CAD did not differ in patients who presented with positive SYNTAX score. This could introduce the notion that SMuRF-less patients represent a heterogeneous population who exhibit larger variation in the severity of CAD (22, 23). Another cohort study of patients with CAD observed lower plaque burden and calcification in SMuRF-less patients, however, there was no difference in the progression of atherosclerotic plaque progression between the two groups (24). These observed differences in CAD severity and atherosclerotic burden of patients without SMuRFs might be attributed to heterogeneous underlying biology, genetic or epigenetic differences, environmental exposures, and interactions with chronic clinical entities such as autoimmune disorders or chronic kidney disease (23). Unfortunately, this study was not empowered to detect any of these differences.

Moreover, our study adds to the growing body of literature that SMuRF-less patients with ACS suffer from excess early all-cause mortality risk (either in-hospital or at 30 days of follow-up) (5–7, 21); yet the long-term post-ACS clinical course did not differ significantly among patients with and without SMuRFs of our study, which also concurs with some of the published reports (7, 17). Interestingly, Figtree et al. showed that the events contributing to higher all-cause mortality in SMuRF-less patients occurred in the first 30 days; yet all-cause mortality remained increased in the SMuRF-less group at a 8-year patient follow-up (7). Nevertheless, a recent study presented discordant outcomes demonstrating that SMuRF-less patients with NSTEMI had lower in-hospital rates of cardiovascular mortality and major adverse events than patients with (9). It has been proposed that this discordance might be due to the underlying etiology of NSTEMI or due to the possible enrollment of patients with milder subclinical forms of SMuRFs across the continuum of SMuRF risk (25).

According to Figtree et al., a major part of observed excess early mortality in SMuRF-less patients could be attributed to arrhythmic causes, since early deaths of STEMI patients were not associated with re-infarctions, strokes, recurrent revascularization procedures, and heart failure-related hospitalizations (7). Hence, the authors supposed that heightened post-MI activity of downstream pathways might increase arrhythmia susceptibility independently of the SMuRFs (7). In addition, SMuRF-less CAD patients seem to have specific challenges concerning the use of guideline-recommended pharmacotherapies early post-MI according to the SMuRF-less status (7, 20). Although these secondary prevention therapies have been developed to target specific risk factors, they have been well-established as pivotal medications with pleiotropic benefits (23, 26). Therefore, these medications should be considered post-ACS regardless the presence of dyslipidemia and hypertension, which is also clear in both American and European guidelines, since they do not differentiate ACS management based on SMuRF-less status (22, 23, 27).

### Limitations and future strategies

Our findings should be interpreted in the context of several limitations. Firstly, this is an observational analysis, prone to bias due to undiscovered confounding, despite the propensity-score-matching approach. Secondly, our data derive from a relatively limited sample of Greek patients presenting with ACS in a PCI-referral hospital and, hence, our analyses – yielding wide CIs of the effect measures – may not apply to other (multi-ethnic) populations. Thirdly, there were no available data on the cause of patients’ death (cardiac or non-cardiac) and, hence, we could not associate the progressively increasing death rate or any death etiology with the SMuRF-less status Furthermore, SMuRFs were defined as binary variables based on clinical diagnoses and accepted cut-off values; yet, DM, dyslipidemia and hypertension might have been also defined as continuous ones to assess the potential gradient of the relative risk. With regard to smoking status, data on previous (ex-) smoking status or the number of pack-years were not available. Data regarding patients’ diet and physical activity were also not available. Lastly, this study did not assess novel known markers of cardiovascular disease such as high-sensitivity C-reactive protein, triglyceride and lipoprotein(a) levels along with novel gene scores.

Nevertheless, this study adds to the existing literature about SMuRF-less status and triggers future efforts for the discovery and further investigation of novel biomarkers [e.g., lipoprotein(a), biomarkers of inflammation, cardiac autonomic dysfunction or clonal hematopoiesis (28–31)] and mechanisms of CAD [coronary artery embolism, vasospasm, and spontaneous coronary artery dissection (32)] beyond SMuRFs. Personalized risk assessment tools including novel cardiovascular risk markers, polygenic risk scores (33) and multi-omic fingerprints (34) seem to be particularly relevant to SMuRF-less patients and might facilitate new preventative strategies and personalized pharmacotherapies (35).

Current guidelines do not specifically address the management of CAD in patients without SMuRFs (23). Enhanced representation of this CAD population in future trials, in conjunction with secondary *post hoc* analyses of existing trials and patient-level meta-analyses, could raise hypotheses and provide significant insights directing clinical pathways for this group of patients. Future larger studies should first validate our findings, before making any implications for different therapeutic management of some SMuRF-less patients with ACS with angiographically less severe CAD disease (zero SYNTAX score) and until then society guidelines should be followed for all patients with ACS, regardless of the SMuRF status.

## Conclusion

In conclusion, SMuRF-less status may be associated with increased odds for non-obstructive CAD, although the overall distribution of SYNTAX score did not differ substantially between patients with and without SMuRFs. Our survival analysis suggests that absence of SMuRFs is significantly linked with increased risk of 30-day all-cause mortality, whereas long-term mortality rates were similar. These findings highlight the need for further research into SMuRF-less status in ACS patients and potential implications on the efficacy of guideline-recommended management, especially during the immediate post-ACS period.

## Data Availability Statement

The raw data supporting the conclusions of this article will be made available by the authors, without undue reservation.

## Ethics statement

The studies involving human participants were reviewed and approved by the Scientific Committee of AHEPA University Hospital (reference number 12/13-06-2019) and by the Directory Board of AHEPA University Hospital (reference number: 17/29-08-2019). The patients/participants provided their written informed consent to participate in this study.

## Author contributions

GSo, GGP, and IF contributed to the conception or design of the work. AP, IF, SZ, EK, NS, DM, and AK contributed to the acquisition, analysis, or interpretation of data for the work. AP, IF, and SZ drafted the manuscript. EK, NS, DM, AK, ID, GGK, and GSi critically revised the manuscript. All authors gave final approval and agreed to be accountable for all aspects of work ensuring integrity and accuracy.

## Conflict of Interest

The authors declare that the research was conducted in the absence of any commercial or financial relationships that could be construed as a potential conflict of interest.

## Publisher’s Note

All claims expressed in this article are solely those of the authors and do not necessarily represent those of their affiliated organizations, or those of the publisher, the editors and the reviewers. Any product that may be evaluated in this article, or claim that may be made by its manufacturer, is not guaranteed or endorsed by the publisher.
