# Supplementary material for: Angiographic severity in acute coronary syndrome patients with and without standard modifiable risk factors
Source: Front Cardiovasc Med. 2022 Jul 22;9:934946. doi: 10.3389/fcvm.2022.934946 (PMC9353176; doi:10.3389/fcvm.2022.934946)
Supplement: Supplementary file 1 [file Data_Sheet_1.docx]

Supplementary Material

# Supplementary Figures and Tables

## Supplementary Figures


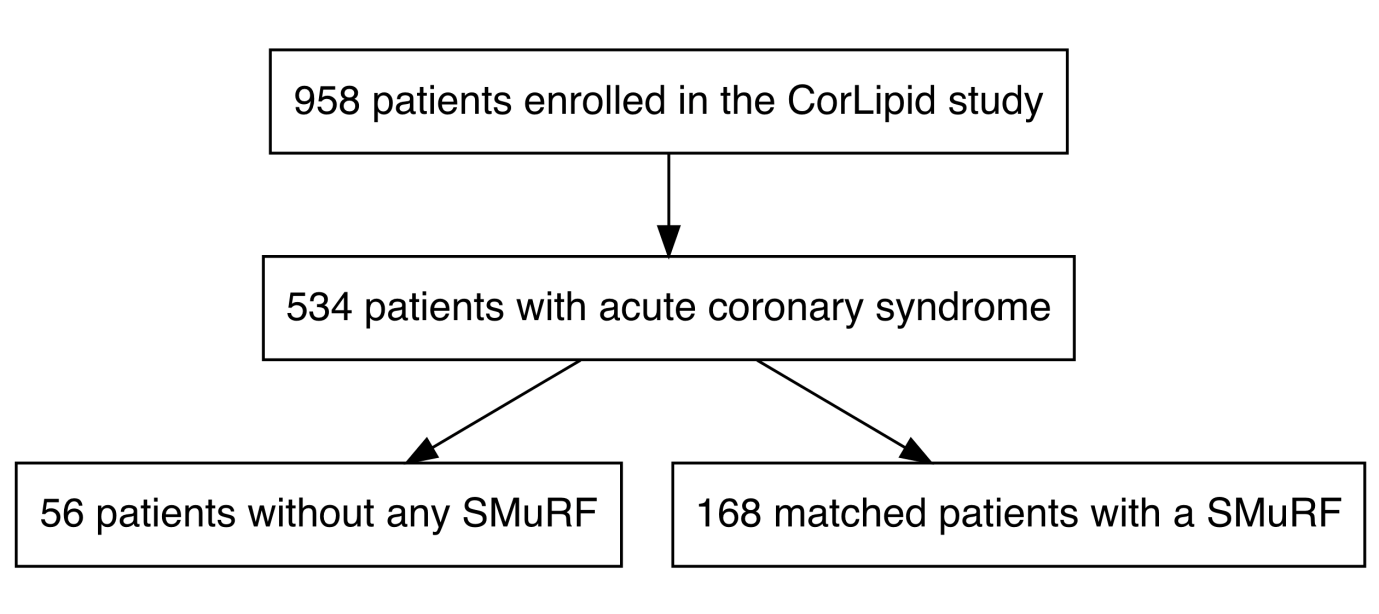


## Supplementary Figure 1. Study flowchart: Of 958 patients enrolled in the CorLipid trial, 56 of them presented with ACS without having any SMuRF and were matched to 168 patients having at least one SMuRF (propensity score matching ratio 1:3).

##
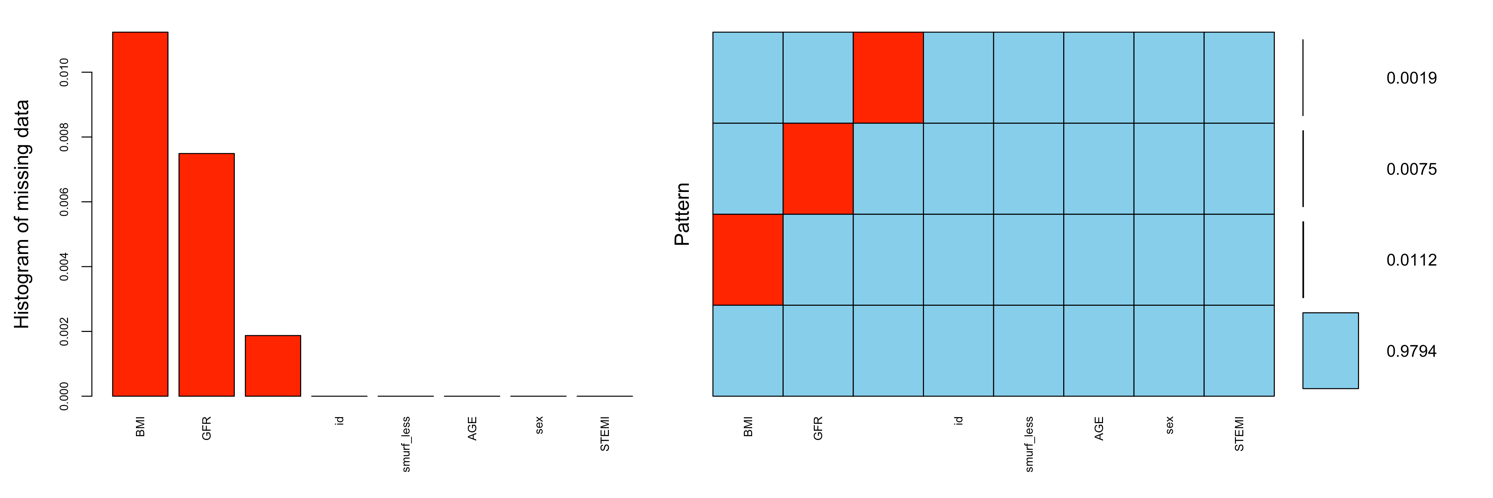
Supplementary Figure 2. Pattern of missing values in the covariates used for propensity score matching, indicating minimal proportion of missing values.

**
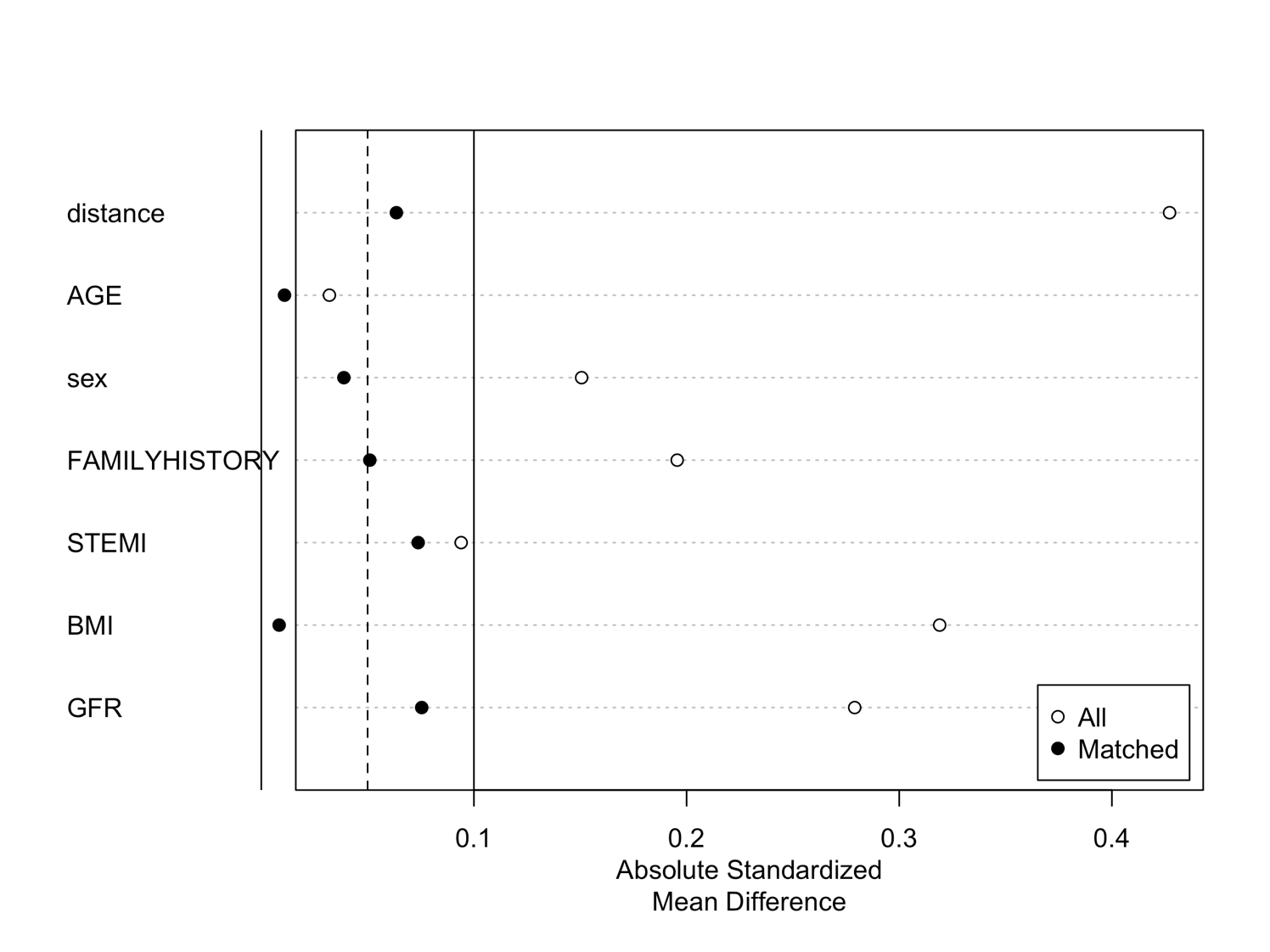
**

**Supplementary Figure 3.** Love plot created for the balance assessment of the propensity score matching baseline covariates. All matched baseline covariates seem to be well-balanced (absolute standardized mean difference <0.1).

**1.2. Supplementary Tables**

**Supplementary Table 1.** Baseline characteristics of the matched population

| **Characteristic** | **Overall**,  N = 224 | **Patients with at least one SMuRF**,  N = 168 | **Patients without SMuRFs**, N = 56 | **p-value***^1^* |
| --- | --- | --- | --- | --- |
| **Age, years, Median (IQR)** | 64 (16) | 64 (15) | 66 (24) | 0.8 |
| **Sex, male, n(%)** | 159(71.0%) | 120(71.4%) | 39(69.6%) | 0.8 |
| **BMI, kg/m2, Median (IQR)** | 26.3 (4.5) | 26.4 (4.3) | 26.2 (5.9) | >0.9 |
| **Hypertension, n(%)** | 104(46.4%) | 104(61.9%) | 0(0.0%) | <0.001 |
| **Diabetes Mellitus, n(%)** | 53(23.7%) | 53(31.5%) | 0(0.0%) | <0.001 |
| **Dyslipidemia, n(%)** | 65(29.0%) | 65(38.7%) | 0(0.0%) | <0.001 |
| **Smoking, n(%)** | 95(42.4%) | 95(56.5%) | 0(0.0%) | <0.001 |
| **Family History of CAD, n(%)** | 29(13.0%) | 21(12.6%) | 8(14.3%) | 0.7 |
| **Previous stroke, n(%)** | 7(3.1%) | 7(4.2%) | 0(0.0%) | 0.2 |
| **PAD, n(%)** | 10(4.5%) | 9(5.4%) | 1(1.8%) | 0.5 |
| **Atrial fibrillation, n(%)** | 20(8.9%) | 13(7.7%) | 7(12.5%) | 0.3 |
| **CKD, n(%)** | 17(7.6%) | 14(8.3%) | 3(5.4%) | 0.6 |
| **COPD, n(%)** | 7(3.1%) | 6(3.6%) | 1(1.8%) | 0.7 |
| **Severe aortic stenosis, n(%)** | 4(1.8%) | 2(1.2%) | 2(3.6%) | 0.3 |
| **Heart failure, n(%)** | 4(1.8%) | 4(2.4%) | 0(0.0%) | 0.6 |
| *^1^*Wilcoxon rank sum test; Pearson's Chi-squared test; Fisher's exact test | | | | |
